# Designing a SPIKES-based protocol for communicating uncertainty in indeterminate thyroid cytology: a mixed-method analysis within a pilot study

**DOI:** 10.1007/s13304-025-02346-3

**Published:** 2025-08-27

**Authors:** Rossella Melcarne, Fabrizio Consorti, Giorgio Grani, Tal Deborah Engel, Eva Iannuzzi, Carla Ammendolia, Laura Giacomelli, Cosimo Durante, Marco Biffoni

**Affiliations:** 1 Department of Translational and Precision Medicine, Sapienza University of Rome, Viale del Policlinico, 155, 00161 Rome, Italy; 2 Department of General Surgery, Sapienza University of Rome, Viale del Policlinico, 155, 00161 Rome, Italy; 3 Department of General and Specialist Surgery, Sapienza University of Rome, Viale del Policlinico, 155, 00161 Rome, Italy

**Keywords:** Risk, Shared decision making, Surgery residency, Thyroid nodules, Patient-centered care, Soft skills

## Abstract

Indeterminate thyroid cytology presents a significant diagnostic challenge, often leading to difficult treatment decisions for both physicians and patients. Effective communication of these uncertainties is crucial, especially in a surgical setting, where decisions can have long-lasting impacts. This is particularly important for younger surgeons who may lack extensive clinical experience. This study aimed to plan, develop, implement, and pilot a communication protocol designed for surgeons, particularly those in training, to navigate uncertainty and build trust with their patients. Additionally, it documented the reactions of patients managed using this approach. Fifty-two patients diagnosed with indeterminate thyroid cytology were enrolled between March 2023 and January 2024 at the Endocrine Surgery Unit of "Azienda Ospedialiera Universitaria Policlinico Umberto I" in Rome, Italy. A communication protocol based on the SPIKES model (Setting, Perception, Invitation, Knowledge, Emotion, Strategy), a six-step protocol for guiding  difficult conversations, was designed to structure discussions around uncertain diagnoses. Patient consultations were conducted in three phases: the initial outpatient visit, pre-surgery admission, and a follow-up one week after surgery. During each encounter, patients' emotional responses were recorded using open-ended questions. To ensure clarity, a Decision Aid (DA) tool was introduced during the K-Knowledge phase of SPIKES, to better explain the risks and treatment options. A mixed-methods approach was used to analyze both qualitative and quantitative data from patient feedback. The adapted protocol was found to be effective in enhancing patient comprehension and emotional response. Approximately 80% of patients reported emotional relief after the first consultation, and by the third encounter, 91% expressed a sense of well-being or trust. The most frequently reported emotional states across all three encounters were serenity, reassurance, and trust. A small subset of patients continued to experience concern or fatigue. Additionally, 96% of participants rated the communication as clear, and 88.5% considered the final therapeutic decision as the correct one. This pilot study suggests that a structured SPIKES-based communication protocol can help address gaps in patient understanding, trust, and satisfaction when managing indeterminate thyroid cytology. Implementing such protocols may streamline communication in surgical settings, improve  patient-centered care, and support physicians, particularly trainees, in managing complex patient interactions.

**Supplementary Information:**

The online version contains supplementary material available at 10.1007/s13304-025-02346-3.

## Introduction

Indeterminate thyroid cytology, a term used to describe thyroid nodules that cannot be definitively classified as benign or malignant through cytological analysis alone, is a common diagnostic challenge [1]. Two frameworks that have proven valuable in these cases are the Bethesda System for Reporting Thyroid Cytopathology (BSRTC) [2] and the Italian Consensus for Thyroid Cytology [3], as they offer standardized guidelines for categorizing thyroid nodule aspirates obtained through fine-needle aspiration (FNA). In the BSRTC, nodules are assigned to “category III” (atypia of undetermined significance) or “IV” (follicular neoplasm). These findings, which are reported in about one third of all thyroid pathological cytology, are associated with expected malignancy rates of 13–30% and 23–34%, respectively [3]. According to the Italian Consensus for Thyroid Cytology [3], they are described as TIR3A and TIR3B, respectively.

These indeterminate diagnoses underscore the need for a more nuanced approach to patient care, balancing the risks of malignancy against the potential harm of unnecessary surgical interventions [4]. The rapidly advancing field of molecular diagnostics continues to refine the accuracy of cytology assessments, allowing for more precise and personalized treatments [5, 6]. While terms like “precision medicine” and “molecular genomics” suggest certainty and the promise of increasingly targeted therapies, the paradox is that with more information comes greater uncertainty [7]. Communicating indeterminate nodule diagnoses is particularly challenging, especially for younger physicians who, with limited clinical experience, may still be honing their strategies for addressing uncertainty with patients [8, 9]. In these borderline cases, a structured communication model could improve the quality of care and foster stronger clinician-patient relationships based on trust and mutual understanding [10–12]. Acknowledging such need, a group of physicians initiated a project to develop and implement a model aimed at effectively managing and embracing uncertainty in clinical surgical practice. Frameworks like Shared Decision Making (SDM) and tools like Decisional Aids (DAs) assist in broadening patient-perceived knowledge and have shown to improve the decision-making process [13]. SDM has demonstrated a reduction in overdiagnosis and unnecessary treatments, aligned care options with a patient's personal preferences, and ensured consistency in care among hospital practitioners [14–16]. Furthermore, SDM reduced patient’s anxiety about their treatment and decisional conflict [16, 17].

In the context of surgery, some suggest that SDM may be ineffective due to the informational nature of surgical consultations compared to the value-based discussions common in medicine [18, 19]. For example, in fields like trauma, the limited time available often precludes thorough discussions of alternative treatments or associated risks and benefits. Yet it is this very imperative  that emphasizes the importance of SDM in surgery: surgical procedures are frequently irreversible and can lead to significant changes in physical capabilities [18, 20, 21]. Therefore, incorporating SDM has shown to be of great significance in considering patient outcomes, particularly when evaluating the long-term benefits regarding operating on uncertain diagnostic findings (for example whether to undergo preventive surgery) [22] and respecting patient autonomy [23].

In this article, we describe the planning, development, implementation, and pilot evaluation of a protocol designed to help surgeons, particularly those in training, communicate uncertainty and build trust with their patients. Specifically, we examine whether a SPIKES-based protocol [24] can be effectively applied to facilitate communication between surgeons and patients with indeterminate thyroid cytology.

## Methods

### Study design

This pilot study employed a mixed-method approach. The data analyzed validated the reaction of patients when their treating doctor communicated a diagnosis of uncertain thyroid nodules using a structured communication protocol. The study was conducted in two phases: protocol planning and development, and evaluation of the designed protocol. Closed-ended questions were used to analyze the frequency of responses. These frequencies were  not intended to provide statistical significance but rather to descriptively illustrate the phenomenon. This manuscript has been prepared in full adherence to the SQUIRE guidelines [25] (Appendix 1).

### Setting

The study was conducted at the “Azienda Ospedaliera Universitaria (AOU) Policlinico Umberto I” - “Sapienza” University of Rome specifically within the “Endocrine Surgery Unit (ESU)”, which is nationally accredited as a Thyroid Surgery Unit. This unit collaborates closely with the “Translational and Precision Medicine Unit” of the same hospital, which is, among the others, dedicated to the diagnostic assessment, surveillance, and treatment of patients with thyroid nodular disease and thyroid carcinomas. Due to this collaboration and the recognized expertise of these units, the volume of patients presenting with uncertain diagnoses is significant. In 2022, approximately 30% of surgical cases managed by the ESU involved patients with a preoperative diagnosis of TIR3, underscoring the substantial prevalence of indeterminate thyroid nodules addressed by the surgical team. The study began in January 2023, with an initial two-month phase (January and February 2023) focusing on protocol development by a team of endocrine surgeons, coordinated by a reviewer with expertise in medical education and communication. Following consensus within the team and additional internal reviews by the ESU, patient recruitment started in March 2023 and continued until January 2024. The data collected during this period was  analysed after the recruitment phase ended.

### Participants

Patients were selected based on a recent diagnosis of indeterminate thyroid nodules (TIR3, SIAPEC 2014) obtained through fine needle aspiration, referred by the endocrinologist to the ESU for assessment for surgery (nodule TIR3B and/or TIR3A repeated and confirmed in ≥ 2 fine-needle aspirations over a period of ≥ 2 years). Patients were excluded if they had coexisting nodules with a malignant cytology pattern (TIR4 and/or TIR5), a personal history of malignant thyroid disease, or if they had already undergone thyroid lobectomy. To minimize selection and communication biases, the study was conducted at a single center -AOU Policlinico Umberto I of Rome, “Sapienza” University -and included only fluent Italian-speaking patients to ensure consistency in communication and uniformity in patient management practices. Patients were referred for consultation with an endocrine surgeon following an initial visit and ultrasound evaluation.

### Protocol planning

#### Literature review

An extensive review of existing guidelines and medical communication protocols was conducted using databases such as PubMed, Scopus, and Web of Science to gather relevant articles published over the last ten years. Several evidence-based communication models exist for breaking bad news or managing uncertainty, including SPIKES, BREAKS, ABCDE, COMFORT, and PEWTER [24, 26–30].

While many of these were developed for oncology or palliative care, we chose SPIKES [24] for its structured yet flexible format, which can be adapted to surgical consultations, and supports both  surgeons and patients during complicated  conversation [24, 31, 32]. Compared to models such as BREAKS or COMFORT, which emphasise relational or family-oriented approaches, SPIKES provides a practical framework that supports the integration of empathic dialogue and decision support -making it particularly suitable for surgical settings involving diagnostic ambiguity [27–29, 32, 33, 34] (Table 1).

**Table 1:**
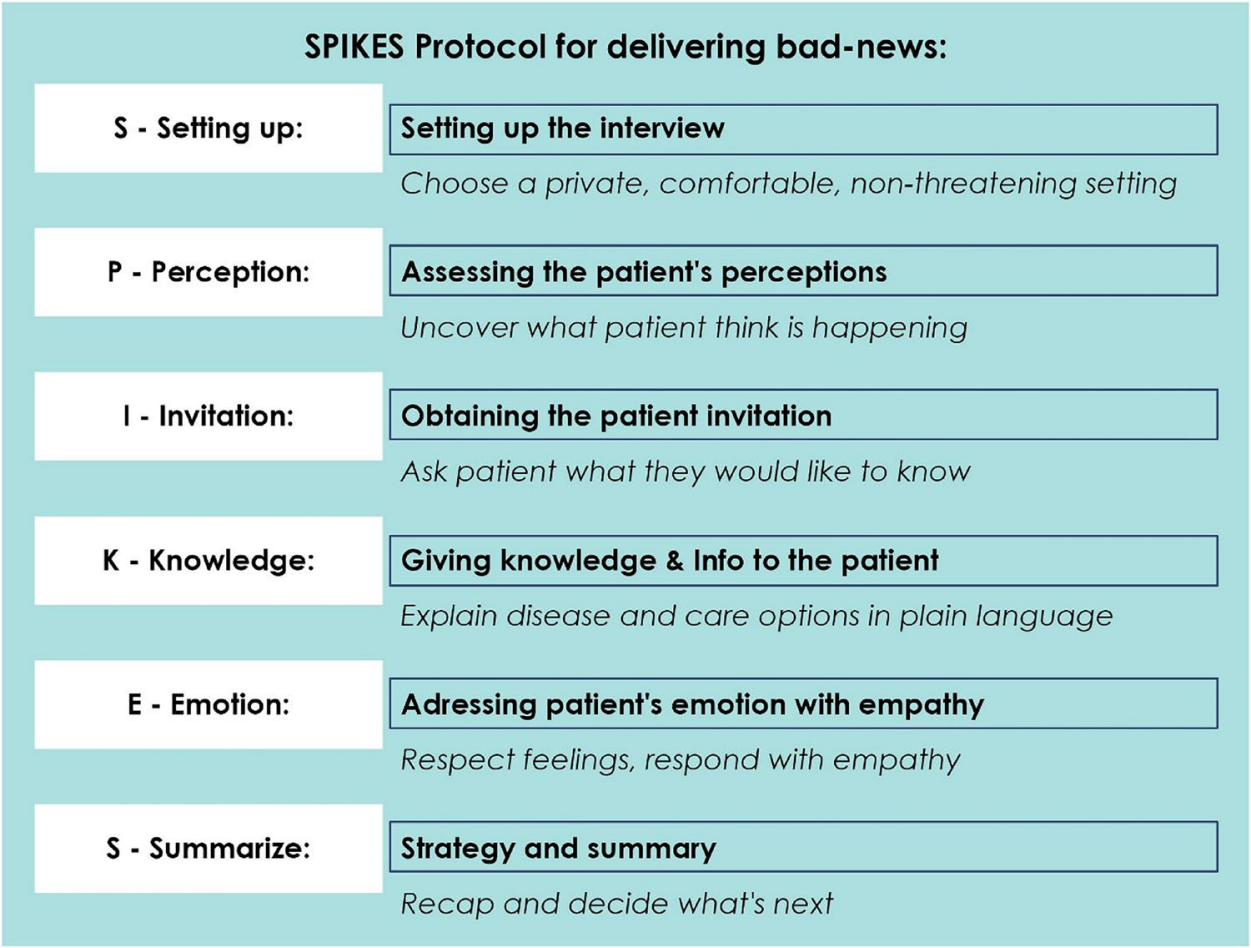
Overview of the SPIKES protocol for breaking bad news in clinical settings. This table outlines the six essential steps of the SPIKES protocol, a structured approach designed to guide clinicians in delivering difficult diagnoses with sensitivity and clarity

### Protocol development (Fig. 1)

**Fig. 1:**
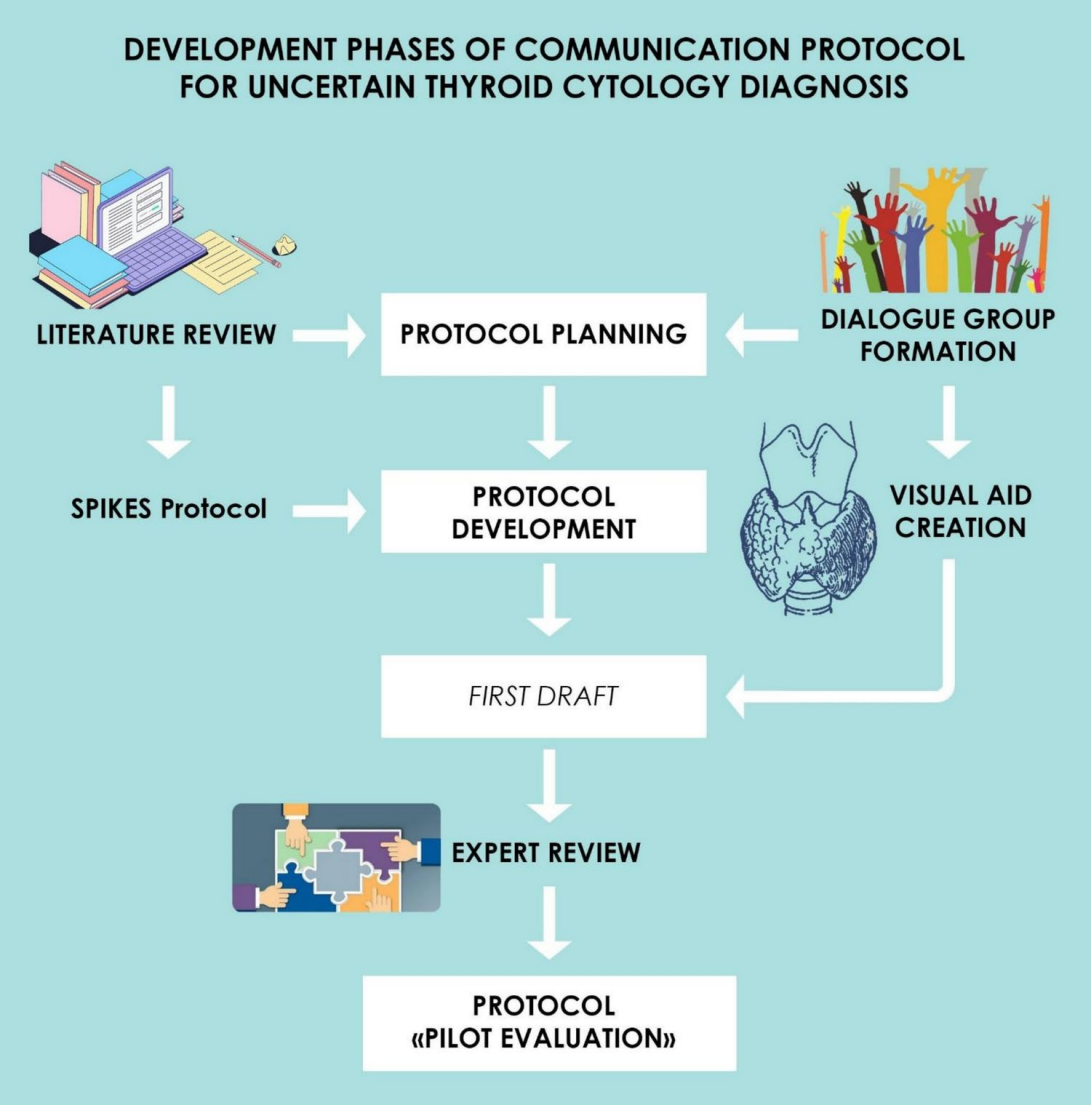
Phases of communication protocol development. This figure outlines the key phases involved in the development of the communication protocol for conveying uncertain thyroid nodule diagnoses

#### Dialogue group

A group named the “Dialogue Group”, consisted  of medical professionals who regularly work with patients with thyroid endocrine disorders, was established to identify key concerns and preferences in communicating uncertain diagnoses. During each discussion, the doctors presented their proposals and insights. At the end of each session, a summary was drafted, outlining the recommendations derived from each participant's proposals.

#### Putting together the protocol

The first protocol draft was established based on literature review and the recommendations from the “Dialogue Group”. This draft included structured guidelines to provide information on the diagnosis of TIR3 thyroid disease, treatment options, and the risks and benefits of each choice, while also addressing patient concerns and doubts. The protocol was designed to assess patients' reactions -including emotional responses- through both open-ended and closed-ended questions, aiming to capture the impact of communicating an uncertain TIR3 diagnosis.

#### Development of the decision aid (DA)

Decision Aid (DA) was developed as a core element of the communication protocol to support patient understanding and shared decision-making. It was integrated into the SPIKES model in the Knowledge section, where it is especially important to clearly convey the concepts of “risk” and “uncertainty”.

Structured as a PowerPoint presentation, the DA combined brief text, charts, and “visual metaphors”, such as a trapdoor filled with black and white balls to represent probabilistic outcomes, or a Likert-style ladder to position perceived risk. These elements aimed to simplify complex concepts and make uncertainty more graspable.

The DA is intentionally designed to be interactive: patients were encouraged to ask questions and reflect during the presentation, enabling a tailored and person-centred dialogue. Full content is available in Supplementary Appendix 2***.***

#### Experts review

The draft protocol was reviewed by experts in endocrine surgery, clinical practice and medical communication to evaluate its clarity, effectiveness, and how the content was perceived by patients. Any feedback received was carefully considered and incorporated into the final version of the protocol.

### Pilot evaluation of the protocol

The revised protocol was first implemented during consultations with fifty-two patients. Two general surgery residents were involved in each consultation: a more advanced resident led the patient interview, while an early-stage resident recorded the patient's responses without actively participating. An experienced surgeon supervised the interactions from a distance, intervening only if significant communication issues arose.

### The communication protocol - protocol steps (Fig. 2)

**Fig. 2:**
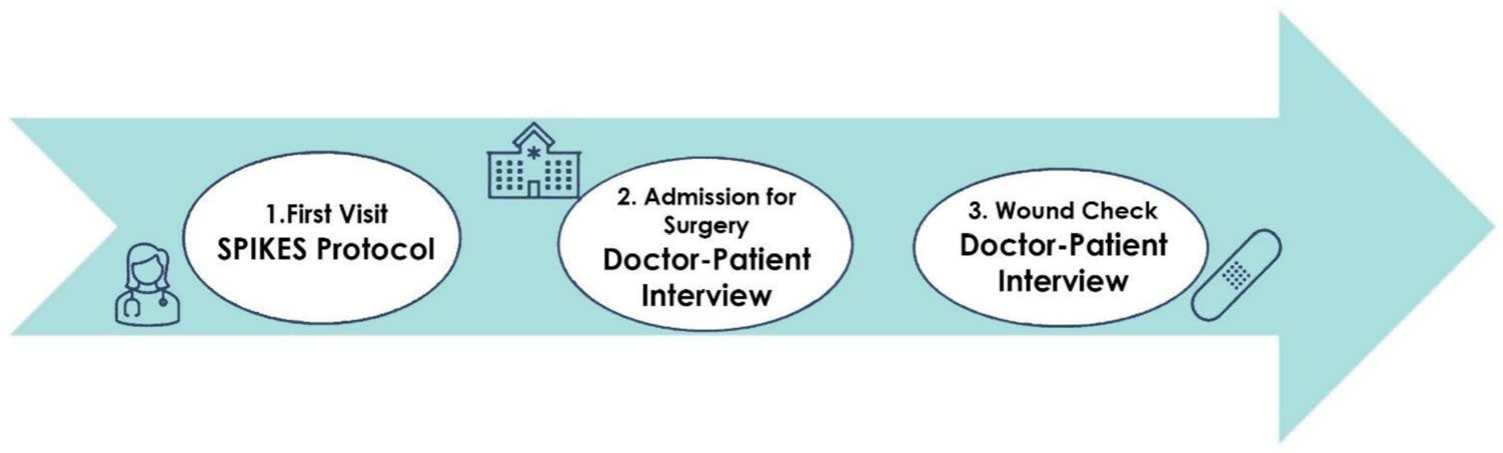
Steps of communication protocol. This figure outlines the key steps of the communication protocol for conveying uncertain thyroid nodule diagnoses

All patients fitting the study criteria were invited to participate in the study. They were also asked for their consent to have an additional physician present in the room, alongside the lead interviewer, who would fully transcribe the patients' responses.

Patient interviews were conducted during three different phases: during the patient's first meeting with the surgeon, the second phase at the time of hospital admission for surgery, and a third after the operation.

Supplementary Appendix 3 includes the full list of topics and questions, organized according to the SPIKES protocol, that were used during the three clinical encounters.

### Evaluation

#### Qualitative and quantitative data analysis

All transcripts of the interviews and field notes taken during the encounters were analyzed.

The qualitative analysis focused on breaking down and classifying the verbal messages into their simplest constituent elements,  breaking down the “communication unit” into “classification units” through a post-hoc approach; that is, the conceptual grid emerged from reading the corpus rather than being pre-established before data analysis (ex-ante).

**Fig. 3:**
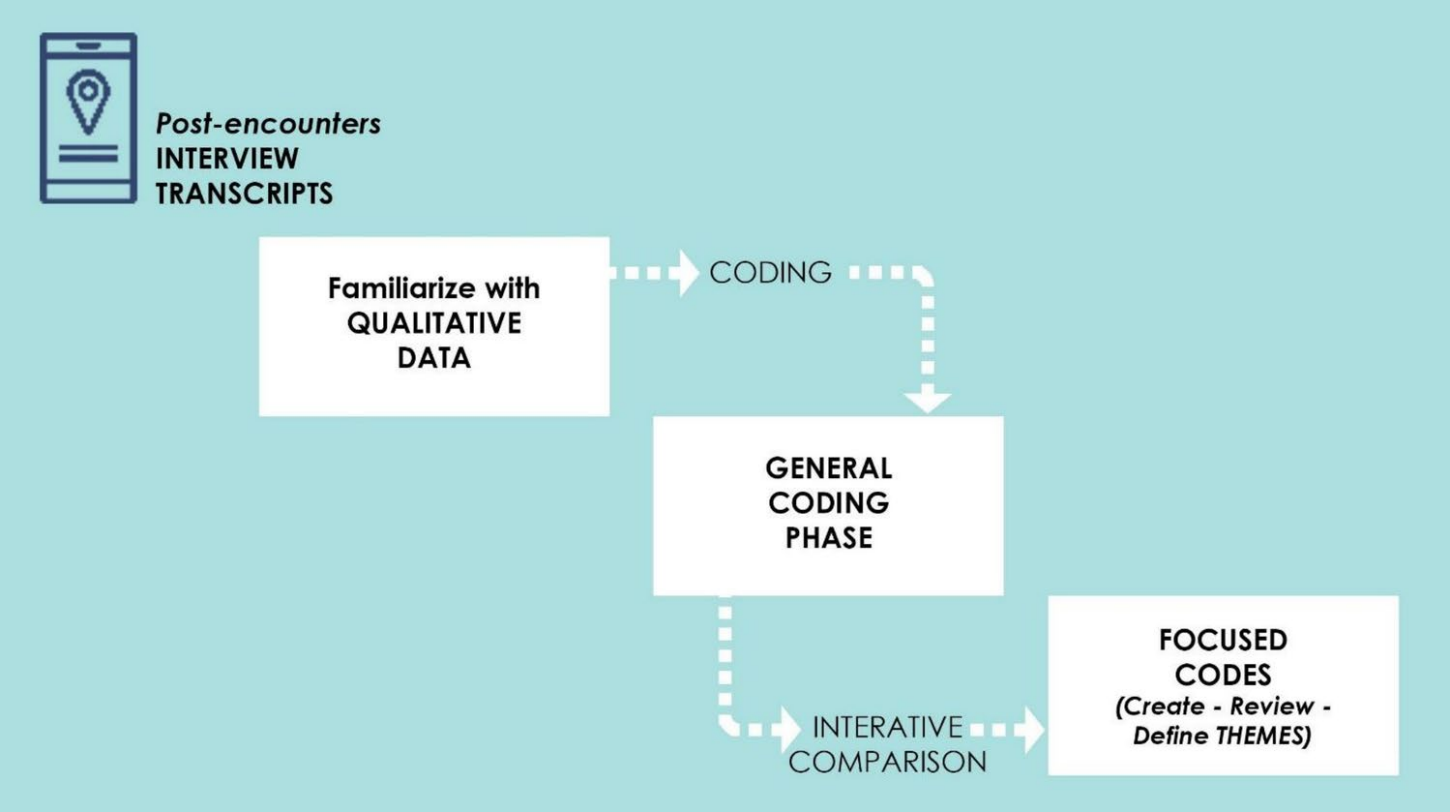
Flowchart illustrates the qualitative data analysis process, outlining the steps from general coding to focused coding and category refinement

The specific procedure consisted of several steps (Fig. 3):

1. *General Coding*: This initial phase involved a comprehensive reading of the responses, during which significant occurrences (i.e., information pertinent to the research question) were identified and selected.
2. *Focused Coding*: In this stage, macro-categories were identified to synthesize the content that emerged from the interview analysis. These macro-categories serve to group related themes and concepts.

To illustrate the distribution of observed phenomena, the frequency of responses was systematically calculated for each thematic category through qualitative content analysis. For closed-ended questions (e.g., Yes/No), response percentages were reported to provide a quantitative summary of participants' answers.

Statistical analysis was intentionally limited to frequency distributions and a chi-square test for categorical comparisons, in line with the exploratory and descriptive aims of the study. Given its formative nature, formal psychometric validation -such as inter-rater reliability or internal consistency- was not applicable at this stage. More comprehensive quantitative analyses will be conducted in future studies involving larger samples.

### Ethical disclaimer

This study was conducted in accordance with standard clinical practice, without the administration of drugs, invasive procedures, or collection of biological samples. The questions posed were those routinely used in clinical consultations, and the model developed is based on pre-existing communication techniques commonly employed in medical settings. As such, the study  carries minimal risk to participants, and no deviation from established ethical guidelines was required. The author(s) selected the following statement: Ethical approval was not required for the studies involving humans because the study participants were co-authors, and their involvement was integral to the research process. As such, they were fully informed about the study's aims and methods. The studies were conducted in accordance with local legislation and institutional requirements. The participants provided their written informed consent to participate in this study. The design adheres to the principles outlined in the Declaration of Helsinki and relevant privacy regulations. While the potential for bias is acknowledged, efforts were made to standardize patient interactions to ensure consistent data collection.

## Results

In this pilot study, conducted from March 2023 to January 2024, fifty-two patients with a diagnosis of indeterminate cytology of the thyroid (TIR 3 category, SIAPEC 2014) were enrolled. Among them, thirty-nine were female and thirteen were male. These patients were referred by their endocrinologists for surgical evaluation at the SEU of the Department of General Surgery, Plastic Surgery, and Orthopedics at AOU Policlinico Umberto I in Rome.

The mean age of the sample was 56.3 years, with a minimum age of 27 and a maximum of 84 years. The mean age for the male sample was 55.7 years, with the youngest patient being 33 years old and the oldest 77 years old; for the female sample, the mean age was 56.5 years, with a minimum age of 27 and a maximum of 84 years.

The analyzed sample had a cytological diagnosis from fine-needle aspiration of the thyroid, categorized as TIR 3 A (low-risk indeterminate lesion) or TIR 3B (high-risk indeterminate lesion) according to the SIAPEC-IAP 2014 classification. Distribution was as follows:

- Thirty-five patients with a TIR 3B cytological diagnosis
- Seventeen patients with a TIR 3 A cytological diagnosis* *Diagnoses were repeated and confirmed in ≥ 2 fine-needle aspirations of the same nodule over a period of ≥ 2 years.

All fifty-two patients underwent surgery, performed by the Endocrine Surgery team at AOU Policlinico Umberto I in Rome:

- Total thyroidectomy: sixteen patients
- Lobectomy: thirty-six patients

The analysis of the final histopathological examination (FHE) post-surgery showed that 48% of the patients had benign thyroid disease (n = 25), while 52% had malignant neoplasms of the thyroid gland (n = 27).

Among the total sample of fifty-two patients, two cases of post-operative complications related to the surgical procedure were reported, specifically one case of transient iatrogenic hypoparathyroidism and one case of dysphonia due to unilateral vocal cord hypomobility.

A synthesis of the thematic categories emerging from patient responses during the first, second, and third clinical encounters is summarized below (Table 2), while the full coding structure and illustrative quotes are reported in Supplementary Appendix 3.

**Table 2:**
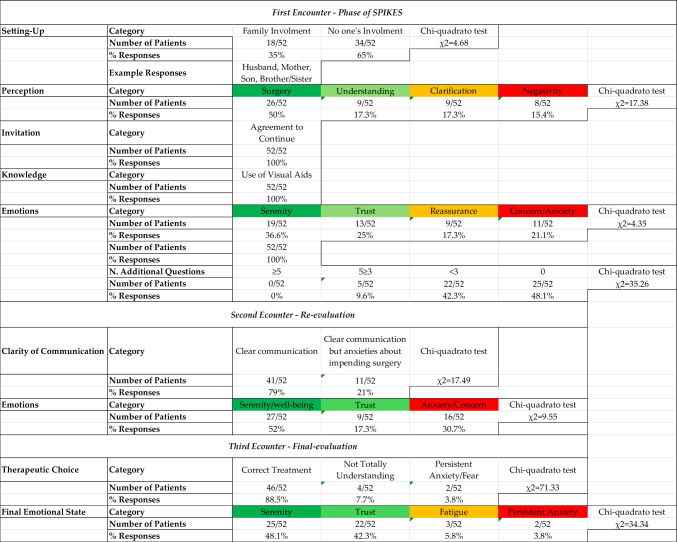
Summary of study results and protocol timeline

## Discussion

This pilot study provided the effectiveness of the proposed communication protocol in conveying treatment choices to patients diagnosed with indeterminate thyroid cytology, integrating SPIKES, shared decision-making principles, and visual support tools to enhance overall patient comprehension of the concept of ‘risk’. This study wishes to highlight how communication is fundamental to healthcare in general [30], and specifically when addressing uncertain diagnoses like indeterminate thyroid cytology (TIR 3) and emphasizing the significance of trust between healthcare providers and patients, understanding, acceptance of the diagnosis, engaging in treatment options, and overall emotional well-being [31].

This adapted protocol represents an innovation in the Italian healthcare system, as it is the first standardized protocol to communicating TIR3 thyroid cytology diagnosis before surgery in Italy to our knowledge, and is also the first attempt to integrate a well-known communication protocol for giving bad news (SPIKES) with visual aids for  communicating uncertainty, framing the process in both pre andpostoperative period. The results show that the communication model used in this protocol was effective in explaining risk information to patients and helped them choose the most suitable treatment based on their needs and preferences. Additionally, the model has created a positive doctor-patient relationship, in a moment that can be confusing and worrying for the patient.

A structured approach, visual support materials, and comprehensive explanations of risks, do improve the communication process, as communicating is more than informing [37]. The results  are patients trusting their doctors and report to be satisfied with their treatment outcome, even in presence of complications [32].

This approach is especially important for young surgeons, who may benefit greatly from a structured framework that guides them in navigating complex patient interactions [38–41].

### Effectiveness of a communication protocol

The communication protocol was structured around the SPIKES model [24], as to allow active participation of patients, and for physicians to create a supportive environment [42].

We noticed the significance of having meetings with patients in a private space, where they could speak freely, the conversation would not be interrupted,  as well as allocating sufficient time [43]. Doctors should assess whether patients can follow and understand the procedures they are about to undergo, and should be given the opportunity to express doubts, fears and personal needs [44]. Clinicians should also look for the “limits of information” patients want to hear, and their preferred mode of communication, without medical paternalism [38]; additionally, doctors should review the information discussed and agreed upon in all future steps for the treatment plan, ensuring mutual understanding [24, 27, 28, 29].

When a serious health problem arises and there are several treatment options, with no single treatment being  preferable to the others, involving the patient in the decision-making process can be greatly beneficial for both the procedure and its outcome [45]. In cases where a decision could lead to a significant impact, patients are often emotional and may need time to process the information [46].

Every decision is influenced by the medical information provided, which involves gathering an enormous amount of data that may not always be fully of use by non- professionals when making critical choices [47]. Another gap to fill is the one between expectations and reality, which can lead to emotional discomfort. Consequently, patient satisfaction largely depends on the degree of congruence between their expectations and the reality they experience [48].

For a doctor-patient relationship to be truly beneficial, it is recommended that doctors share not only the potential therapeutic outcomes but also their doubts and uncertainties. This approach acknowledges that while some patients are motivated to make decisions when faced with uncertainty, others may become anxious and find it difficult to decide [43].

### First encounter

During the first encounter, most patients were willing to involve a family member in the conversation, underscoring the importance of support systems in managing medical communication. Responses to the question about their understanding of the pathology were categorized into four domains: “Surgery”, “Clarification”, “Understanding”, and “Negativity”. This categorization revealed varying levels of patient comprehension and anxiety, with a significant number expressing the need for further clarification and reassurance [22].

Most patients'assessment of risks is not scientifically based [22]. While physicians associate the term"risk"with statistical probability, patients may interpret it as"danger” equating it with life-threatening outcomes, especially when conveyed by a surgeon. To address this discrepancy, we introduced a visual Decision Aid (DA) during the “Knowledge” phase of the SPIKES protocol to help explain the concept of “risk of malignancy”. Although visual formats are recommended for communicating probabilities [44, 46], verbal descriptions remain predominant in clinical encounters [42]. Probability terms such as “possible”, “may”, and “uncertain” are inherently vague [43, 50]; nevertheless, individuals tend to translate these into binary interpretations when making decisions [48].

Our study did not aim to assess the DA in isolation but rather the preliminary feedback, that suggested an improvement in engagement and comprehension. We believe future studies using validated evaluation tools (e.g., Likert scales) are needed to quantify its impact and its reproducibility in broader clinical settings.

### Emotional responses

Communication between physicians and patients goes beyond the mere exchange of information. It involves the nuanced “therapeutic alliance”, sharing thoughts, emotions, and decisions [42]. Poor communication can have detrimental effects, such as the “nocebo” phenomenon, where negative expectations lead to adverse outcomes [51–53]. Historically, the physician–patient relationship was characterized by a paternalistic approach, with patients relying on physicians for unilateral decision-making. This dynamic began to change significantly with the introduction of the Nuremberg Code in 1946 and the Patient's Bill of Rights in 1973 [48]. Today’s medical practice focuses on dialogue, patient autonomy, and collaborative care, highlighting the importance of communication as a tool for both healing and empowerment [52, 53].

In our study, emotional responses were categorized into “Serenity”, “Reassurance”, “Anxiety”, and “Trust”. The distribution of these responses indicated that while many patients felt reassured and trusting after the first conversation, the rest remained anxious, pointing out the ongoing need for empathetic communication and support throughout the entire treatment process. In a state of hypervigilance, with neural activation [54] and limbic system [55]. Anxiety about making the “right” decision may cause the patient to prefer delegating the decision-making responsibility to the physician [45].

Examining the responses received after risk communication by the healthcare provider regarding the patient's emotional state (‘E’ for ‘Emotion’ – See SPIKES) approximately 79% of patients reported a state of “emotional well-being”, spanning the categories of “TRUST, RELAXATION, and SERENITY”. The remaining 21% of patients reported feeling frightened or experiencing anxiety/agitation after the medical communication.

### Second encounter

The second encounter took place the day before surgery. Most patients (96%) found the information provided to be clear, and a substantial proportion (69%) reported feeling confident and calm about the procedure, highlighting the role of ongoing, clear communication in reducing patient anxiety and building a strong therapeutic relationship.

### Third encounter

The final encounter aimed to assess patients' satisfaction with their treatment decisions. An absolute majority (88.5%) felt that the chosen treatment was the correct one, with most expressing confidence and calmness. On the other hand, a small group reported ongoing fear or incomplete understanding, indicating gaps that could  present  opportunities for refining the communication process in the future.

Moreover, the persistence of anxiety observed in a minority of patients even after surgery suggests that decision-making under uncertainty may be influenced by deeper psychological constructs. The Health Belief Model (HBM), for instance, could help explain how patients perceived the severity of their illness, perceived benefits versus barriers to treatment, and residual doubts about the chosen strategy might impact their emotional state over time [56]. Future refinements of this communication protocol could include psychological support, particularly for patients who continue to experience decisional uncertainty or treatment-related stress despite prior clarification and shared decision-making [56, 57].

### Trust as a cornerstone of therapy

During the initial consultation, nearly all patients asked few or no questions about their condition (twenty-two asked a few questions; twenty-five asked none). Most queries concerned post-surgical logistics, such as hospital stay duration, return to work or sports, and stitch removal. This pattern suggests that pre-surgical communication was insufficient, leaving patients under- informed.

In the final summary of our protocol, all participants reported satisfaction with their treatment choice. However, four patients -though generally satisfied- expressed doubts or concerns. Notably, two of these patients, who had not fully grasped the surgical risks preoperatively, only appreciated the significance of complications after experiencing them. These were the only patients in the study who developed postoperative complications: transient hypocalcemia causing paresthesia and transient recurrent nerve injury. This underscores the importance of encouraging patients to ask questions and seek clarification, particularly regarding potential side effects, so they feel reassured and equipped to manage complications. Well-informed patients are more likely to make autonomous decisions rather than passively accept medical recommendations [52, 53, 58].

Explanations often emphasize positive aspects while downplaying risks or side effects. A structured communication protocol can offer a realistic view of disease progression and achievable outcomes, enabling patients to manage risks rather than merely receive information passively [22, 59, 60].

Modern medicine relies on empirical data and statistical analysis to support diagnostic and therapeutic recommendations [7, 61, 62]. Yet in the face of uncertainty, both surgeons and patients may feel unsettled. Surgeons, accustomed to data-driven decisions, may hesitate to disclose uncertainty, fearing it could confuse patients or complicated decision-making. Meanwhile, patients often seek clear guidance and may struggle with ambiguous information. Rather than simply providing more data, patients need interpretation to understand which option best aligns with their values and circumstances [61, 62].

These findings highlight the need for resources and training that enable surgeons to support patient autonomy. Encouraging patients to ask questions, and responding effectively, is essential to fostering shared decision-making [63].

### Practical implications

The significance of a standardized communication model in high-risk situations lies in the inherent delicacy and complexity of these scenarios, which demand clear and effective interaction between physician and patient [56]. Harnessing a model of standardized protocols in conveying uncertain diagnoses confers numerous benefits, including facilitation of the decision-making process and alleviation of emotional strain [27, 40, 57], important for young surgeons, that due to age, experience and preparation were not necessarily  ecquipped for such challenge [22, 64, 65]. Surgeons typically develop their communication skills through professional experience, as formal training in this area during general surgery residency is often inadequate [38, 39, 64, 65]. The focus is usually on technical training and achieving a broad range of surgical experience [63–65]. Nonetheless, surgery involves making critical decisions that can have a profound impact on patients’ lives. Communicating iatrogenic complications or discussing treatment options for uncertain diagnoses involves challenging conversations [15]. These discussions may include complex family dynamics and ethical issues, requiring both sensitivity and mature judgment. This can lead surgeons to grapple with a sense of “failure” (e.g., not being able to prevent all risks). Consequently, communicating, especially when there is uncertainty in the diagnosis, is a significant source of stress for both surgeons and patients, as well as their families. Systematic and comprehensible information delivery reduces ambiguities and errors; by adhering to a protocol, even less experienced physicians can ensure that critical information is neither omitted nor irrelevant details included [44, 57, 66], guaranteeing that every patient receives consistent information and care.

Adapting to a standardized protocol also streamlines the communication process, making it more efficient and saving time for both physicians and patients, an advantage particularly valuable in high-volume settings like surgical departments [22]. Moreover, communicating uncertain diagnoses can be emotionally challenging for both parties. Protocols offer tools and guidelines for managing patients' emotional reactions while also helping physicians handle these situations with greater composure and professionalism [47].

### Limitation of the study

This study has several limitations. First, the small sample size, and second, the single-center data collection, both typical characteristics of a pilot study. The specific participant selection may introduce selection bias and limit the generalizability of the findings. For example, the study was conducted in a specialized thyroid oncology center in Rome and involved patients specifically diagnosed with indeterminate thyroid cytology; therefore, the effectiveness and adaptability of the proposed protocol remain uncertain in different contexts, such as diverse healthcare environments and patient populations.

Additionally, no methodological adjustments, such as protocol refinements based on patient feedback—were implemented to reduce variability and enhance consistency. Frequency distributions were included to provide a preliminary insight into communication-related phenomena; however, statistical significance was not calculated, as hypothesis testing was not among the study's primary objectives.

This approach aimed to offer a descriptive overview of the data distribution and to support hypothesis generation and sample size estimation for future comparative studies. These studies should aim to expand the sample size, including multicenter recruitment, and assess the tool’s performance through more robust quantitative analyses and formal validation strategies.

Furthermore, the impact of the Decision Aid was not formally evaluated using validated psychometric instruments, as it was primarily designed to support the overall communication process by conveying the notion of “possibility” and contextualizing the proposed treatment strategy. This remained the main focus of our investigation.

## Conclusion

In conclusion, this pilot study demonstrates the potential benefits of a structured communication protocol in managing patients with indeterminate thyroid cytology. By improving understanding and emotional well-being, such protocols can play a crucial role in patient-centered care. Future studies should aim to further refine these communication strategies and explore their applicability across different patient populations and clinical settings. Effective communication is an investment. Time spent on communication is time spent on care [67].

## Supplementary Information

Below is the link to the electronic supplementary material.

Supplementary file1 (PDF 65 KB)

Supplementary file2 (XLSX 14 KB)

## Data Availability

The data that support the findings of this study are not publicly available due to privacy or ethical restrictions, but are available from the corresponding author upon reasonable request.
