# Supplementary material for: Designing a SPIKES-based protocol for communicating uncertainty in indeterminate thyroid cytology: a mixed-method analysis within a pilot study
Source: Updates Surg. 2025 Aug 27;78(1):75–87. doi: 10.1007/s13304-025-02346-3 (PMC12909332; doi:10.1007/s13304-025-02346-3)
Supplement: Supplementary file 1 — Supplementary file1 (PDF 65 KB) [file 13304_2025_2346_MOESM1_ESM.pdf]

Strumento di comprensione del rischio di un nodulo della tiroide a citologia indeterminata

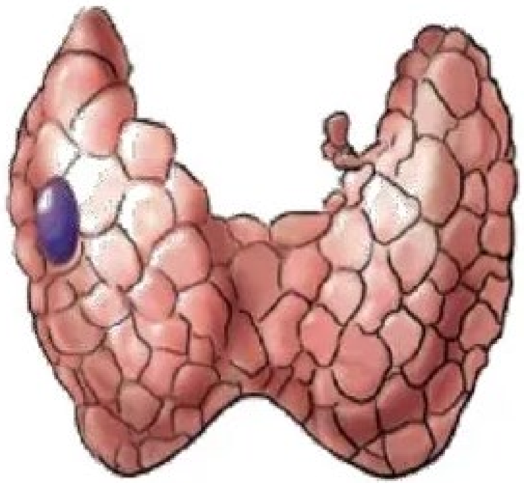

I noduli della ghiandola tiroide sono frequenti

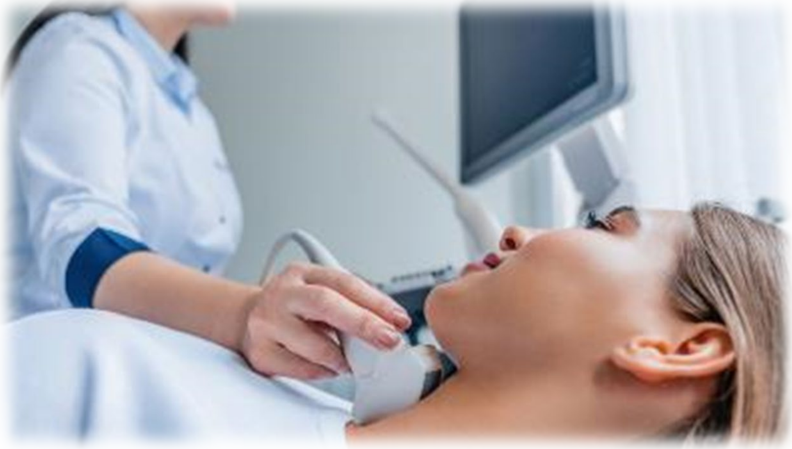

L'ecografia della ghiandola tiroide può rilevare noduli nella popolazione fino al 70% dei casi

Strumento di comprensione del rischio di un nodulo della tiroide a citologia indeterminata

Localizzazione

Dimensioni

Sintomi

Storia familiare

Test genetici

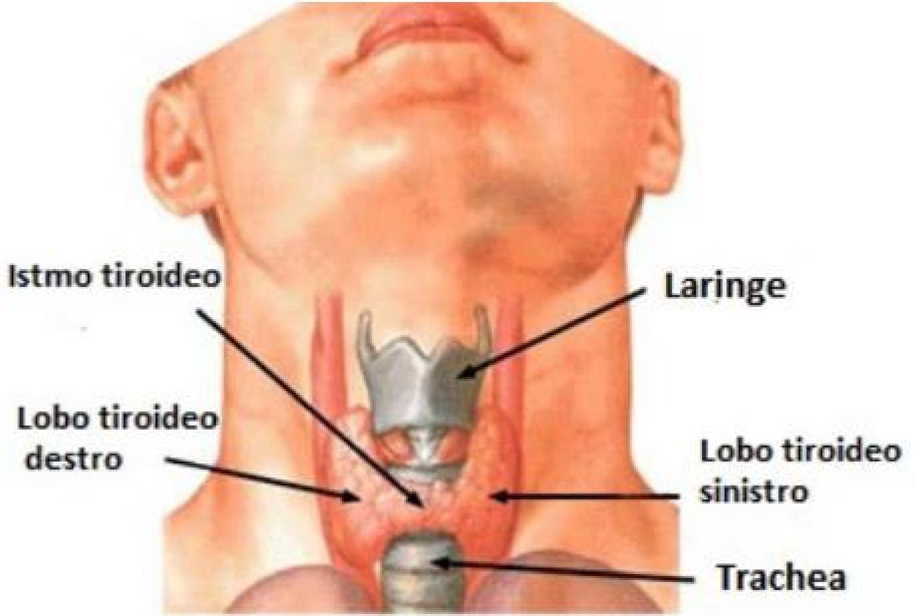

Strumento di comprensione del rischio di un nodulo della tiroide a citologia indeterminata

*Nodulo tiroideo a citologia indeterminata Tir3 (SIAPEC 2014)

**Alterazioni genetiche identificate

Strumento di comprensione del rischio di un nodulo della tiroide a citologia indeterminata

Qual è la possibilità che questo nodulo sia maligno?

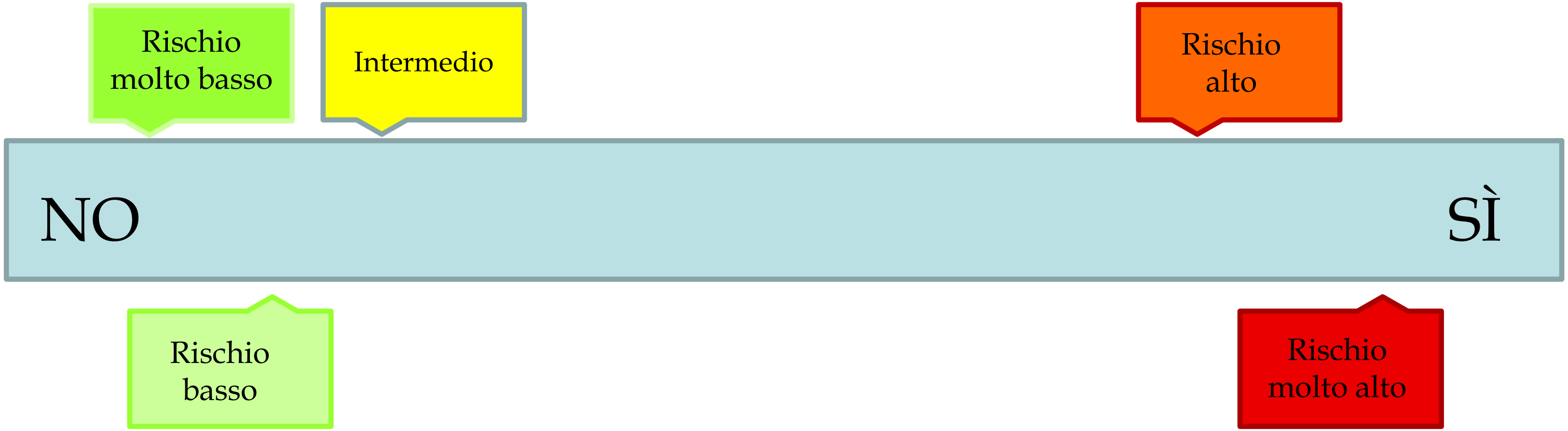

Qual è la possibilità che questo nodulo sia maligno?

Nodulo solido ipoecogeno con:

- Microcalcificazioni
- Margini irregolari
- Estensione extratiroidea
- Aspetto «più alto che largo»
- Rima calcifica interrotta
- Linfonodi sospetti

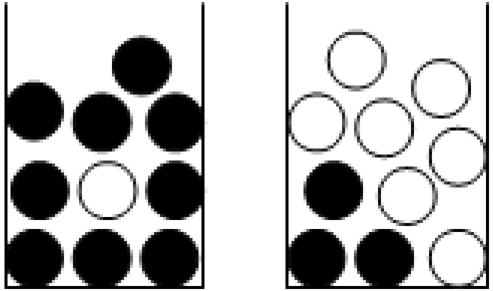

Strumento di comprensione del rischio di un nodulo della tiroide a citologia indeterminata

Cosa fare?

- Monitorare il nodulo

- Chirurgia

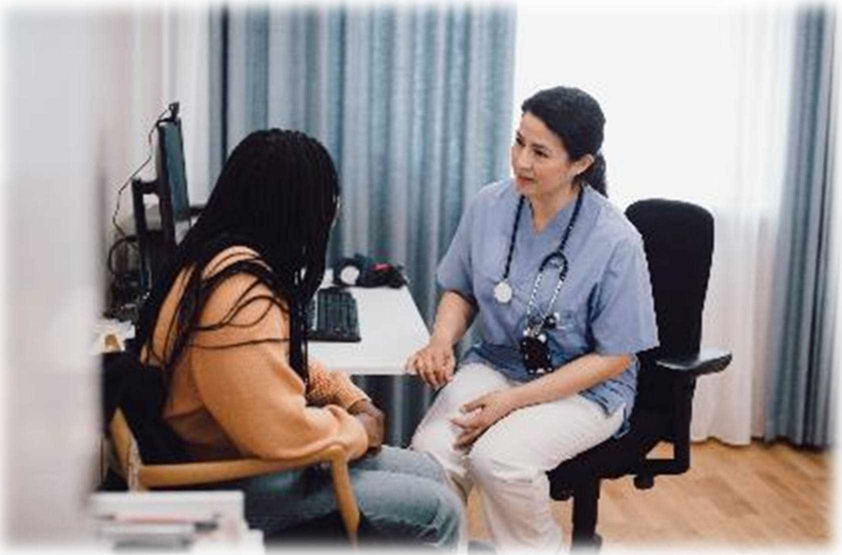

Strumento di comprensione del rischio di un nodulo della tiroide a citologia indeterminata

Cosa fare?

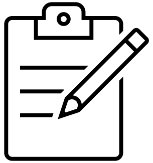

Monitorare il nodulo

- Controlli ecografici e di laboratorio seriati
- Possibilità di dover ripetere agoaspirato del nodulo tiroideo

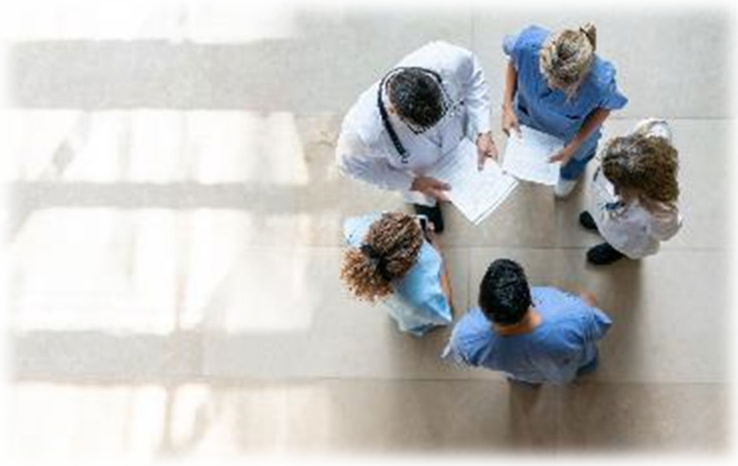

Strumento di comprensione del rischio di un nodulo della tiroide a citologia indeterminata

Cosa fare?

Chirurgia

- Loboistmectomia della ghiandola tiroide
- Tiroidectomia totale

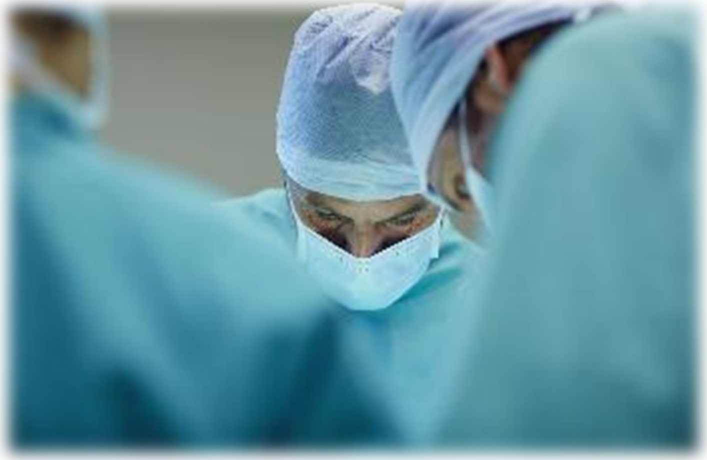

Cosa fare?

Chirurgia

- terapia ormonale sostitutiva –obbligatoria se tiroidectomia totale-
- cicatrice chirurgica sul collo
- possibile cambiamento della voce di tipo temporaneo (8 su 100) o permanente (0,8 su 100)
- possibile riduzione dei livelli di calcio nel sangue temporanea (8 su 100) o permanente (1,8 su 100)
